# Prevention of bronchial hyperreactivity in a rat model of precapillary pulmonary hypertension

**DOI:** 10.1186/1465-9921-12-58

**Published:** 2011-04-27

**Authors:** Walid Habre, Gergely Albu, Tibor Z Janosi, Fabienne Fontao, Britta S von Ungern-Sternberg, Maurice Beghetti, Ferenc Petak

**Affiliations:** 1 Pediatric Anesthesia Unit, Geneva Children's Hospital, University Hospitals of Geneva, 6, Rue Willy Donze, CH-1205, Geneva, Switzerland; 2 Anesthesiological Investigations Unit, University Hospitals of Geneva, 1 Rue Michel Servet, CH-1205, Geneva, Switzerland; 3 Pediatric Cardiology Unit, Department of Pediatrics, Geneva Children's Hospital, 6, Rue Willy Donze, CH-1205, Geneva, Switzerland; 4 Department of Medical Physics and Informatics, University of Szeged, 9 Koranyi fasor, H-6720, Szeged, Hungary

## Abstract

**Background:**

The development of bronchial hyperreactivity (BHR) subsequent to precapillary pulmonary hypertension (PHT) was prevented by acting on the major signalling pathways (endothelin, nitric oxide, vasoactive intestine peptide (VIP) and prostacyclin) involved in the control of the pulmonary vascular and bronchial tones.

**Methods:**

Five groups of rats underwent surgery to prepare an aorta-caval shunt (ACS) to induce sustained precapillary PHT for 4 weeks. During this period, no treatment was applied in one group (ACS controls), while the other groups were pretreated with VIP, iloprost, tezosentan via an intraperitoneally implemented osmotic pump, or by orally administered sildenafil. An additional group underwent sham surgery. Four weeks later, the lung responsiveness to increasing doses of an intravenous infusion of methacholine (2, 4, 8 12 and 24 μg/kg/min) was determined by using the forced oscillation technique to assess the airway resistance (Raw).

**Results:**

BHR developed in the untreated rats, as reflected by a significant decrease in ED_50_, the equivalent dose of methacholine required to cause a 50% increase in Raw. All drugs tested prevented the development of BHR, iloprost being the most effective in reducing both the systolic pulmonary arterial pressure (Ppa; 28%, p = 0.035) and BHR (ED_50 _= 9.9 ± 1.7 vs. 43 ± 11 μg/kg in ACS control and iloprost-treated rats, respectively, p = 0.008). Significant correlations were found between the levels of Ppa and ED_50 _(R = -0.59, p = 0.016), indicating that mechanical interdependence is primarily responsible for the development of BHR.

**Conclusions:**

The efficiency of such treatment demonstrates that re-establishment of the balance of constrictor/dilator mediators via various signalling pathways involved in PHT is of potential benefit for the avoidance of the development of BHR.

## Background

There has recently been substantial progress in the development of new therapeutic strategies for the management of patients with pulmonary hypertension (PHT) [1-5]. The improvements are based on a better understanding of the mechanisms involved in the development of PHT. These treatment strategies are based on the recognition that a key role is played in the modulation of the tone of the smooth muscle cells in the pulmonary vasculature by an imbalance between the vasoactive constrictor and proliferative mediators (endothelin-1 (ET-1), substance P and angiotensin II) and the vasorelaxing and antiproliferative mediators (adrenomedullin, vasoactive intestinal peptide (VIP), prostacyclins (PCs) and nitric oxide (NO)) [6]. The bronchoactive potential of these peptides has been recognized as the major cause of the lung function deterioration [1,2,7-10].

We earlier reported a lung function impairment in a reproducible model of precapillary PHT following the creation of a shunt between the abdominal aorta and the vena cava in rats [11]. We also demonstrated that precapillary PHT leads to the development of bronchial hyperresponsiveness (BHR) to methacholine subsequent to the altered mechanical interdependence between the pulmonary vasculature and the respiratory tract. Although novel strategies are available for the treatment of pulmonary vascular diseases, no studies have yet characterized how the adverse pulmonary consequences of these clinically important pulmonary vascular abnormalities can be prevented. Accordingly, in the present study we set out to explore the efficiency of treatment strategies designed to prevent the adverse changes in the lung function and bronchial responsiveness by acting on the imbalance between the vasoactive constrictor-proliferative and vasorelaxing-antiproliferative mediators.

## Methods

### Animal preparations

The experimental protocol was approved by the Experimental Ethics Committee of the University of Geneva and the Animal Welfare Committee of the Canton of Geneva. Fifty-six adult male Sprague-Dawley rats (weighing 312-382 g) were anaesthetized by an intraperitoneal injection of pentobarbital (70-90 mg/kg of a 50 mg/ml solution). Tracheal intubation was achieved with a polyethylene cannula (16-gauge, Braun, Melsungen, Germany) and the rats were mechanically ventilated with a tidal volume of 7 ml/kg body weight, a positive end-expiratory pressure of 2.5 cm H_2_O, and a respiratory rate of 70-80/min (model 683, Harvard Apparatus Co Inc., South Natick, MA, USA). Anesthesia was maintained with pentobarbital administered intravenously every 40 min (5 mg/kg). The femoral vein was cannulated for drug delivery. The airway pressure, ECG and rectal temperature were monitored continuously by a data collection and acquisition system (Biopac, Santa Barbara, CA, USA). Fentanyl was administered intravenously (15 μg/kg) to ensure adequate analgesia before the administration of pancuronium intravenously (0.4 mg/kg) to facilitate forced oscillatory measurements. To ensure adequate postoperative analgesia, buprenorphine (0.5 mg/kg) was injected subcutaneously before emergence from anesthesia and again 18 h after surgery.

### Induction of precapillary pulmonary hypertension

The surgical induction of precapillary PHT was performed as described in detail previously [11-13]. Briefly, a midline abdominal incision was made in a sterile manner and the infra-renal portions of the abdominal aorta and inferior vena cava were exposed at the site where the two vessels share a common fascia. An aorto-caval shunt (ACS) was then prepared by introducing a needle between the two vessels, followed by application of an aortic adventitial suture. In the rats that received various treatments (see below), an osmotic intraperitoneal pump (Alzet model 2ML4, Alza corporation, Palo Alto, USA) was implanted intraperitoneally into the lower abdomen in order to allow continuous delivery of the different drug medications during 4 weeks. The abdomen was closed in a sterile manner and a long-acting local anesthetic (bupivacaïne 0.25%, 0.8-1.2 ml) was infiltrated around the surgical wounds.

### Treatments, protocol groups

**Figure 1:**
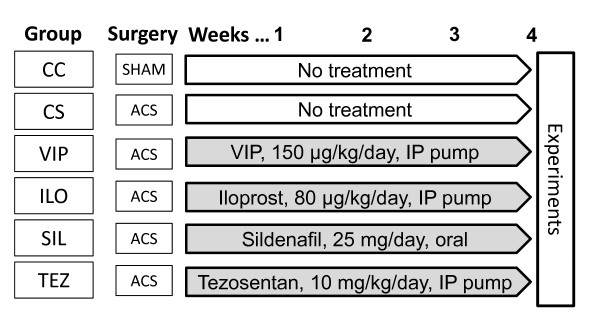
**Experimental protocol**. Zrs: forced oscillatory recordings of the respiratory input impedance. MCh: methacholine.

After creation of the ACS, the animals were randomized to be included in one or other of the groups detailed below (Figure 1). The route of administration of the treatment agents and the doses were chosen on the basis of their pharmacokinetic properties and the manufacturers' recommendations.

#### Group CC (n = 13)

These control rats underwent the surgical procedure as detailed above, but without preparation of the ACS. A subgroup (n = 7) of these animals underwent sham surgery and implementation of an intraperitoneal pump for the continuous delivery of normal saline in the 4-week period, while the remaining rats were not implanted with the pump (n = 6). Since there was no difference between these sham subgroups in the data relating to any of the parameters measuring hemodynamics or respiratory mechanics, their data were pooled for further analyses.

#### Group CS (n = 10)

An ACS was prepared in these animals, but no subsequent drug treatment was applied.

#### Group VIP (n = 8)

To test the VIP pathway in affecting PHT and the subsequent BHR, rats with an ACS received VIP 150 μg/kg/day, administered via the intraperitoneal pump (2.5 μl/h) for 4 weeks.

#### Group ILO (n = 10)

To study the PC pathway in influencing PHT and the subsequent BHR, rats with ACS received iloprost 80 μg/kg for 4 weeks via the intraperitoneal pump (5 μl/h).

#### Group SIL (n = 7)

To investigate the role of NO in the precapillary PHT and the subsequent BHR, ACS preparation was followed by a 4-week treatment with sildenafil administered orally (25 mg/day dissolved in 1.5 ml saline).

#### Group TEZ (n = 8)

To explore how the ET-1-related mechanisms affect the precapillary PHT and the subsequent BHR, rats with an ACS were treated for 4 weeks with the combined ET_A_-ET_B _receptor blocker tezosentan (10 mg/kg/day) administered via an intraperitoneal pump (2.5 μl/h).

### Measurements of end-expiratory lung volume (EELV)

EELV measurements were accomplished in all groups with a body plethysmograph, as detailed previously [14]. Briefly, the trachea was occluded at end expiration until 3 or 4 spontaneous inspiratory efforts had been generated by the animal in the closed box. The changes in tracheal pressure and plethysmograph box pressure during these manoeuvres were recorded, and EELV was calculated by applying Boyle's law to the relationship between the tracheal pressure and the box pressure after correcting for the box impedance [15].

### Forced oscillatory measurements

The contributions of the airway and tissue mechanical properties to the total respiratory resistance were estimated by the forced oscillation technique by measuring the mechanical impedance of the respiratory system (Zrs), as described in detail previously [16,17]. Briefly, the tracheal cannula was connected from the respirator to a loudspeaker-in-box system at end-expiration. Zrs was measured with the wave-tube technique by introducing pseudorandom forced oscillations at frequencies between 0.5 and 21 Hz at end-expiration. Two identical pressure transducers (Model 33NA002D, ICSensors, Malpitas, CA, USA) were used to measure the lateral pressures at the loudspeaker and at the tracheal end of the wave-tube, and Zrs was calculated as the load impedance of the wave-tube by using fast Fourier transformation [16].

To separate the airway and tissue mechanics, a model containing a frequency-independent resistance (Raw) and inertance (Iaw) in series with a constant-phase tissue model [18] including tissue damping (G) and elastance (H) was fitted to the Zrs spectra by minimizing the differences between the measured and modelled impedance values:

where j is the imaginary unit, is the angular frequency (2f) and = 2/arctan(H/G). When this model is fitted to Zrs spectra, the parameter Raw is primarily related to the overall airway geometry, as the contribution of the chest wall to the frequency-independent Newtonian resistance is minor [19]. Similarly, the inertia of the gas in the airways predominates in the parameter Iaw [19].

### Measurement of pulmonary haemodynamics

The pulmonary arterial pressure (Ppa) was measured by introducing a small catheter (polyethylene tubing, ID 0.88 mm, Portex, Hythe, GB) into the pulmonary artery via the jugular vein beforethe animals were sacrificed. This measurement was technically acceptable only in subgroups of the main study groups, involving 7, 10, 4, 5, 2 and 6 rats in Groups CC, CS, VIP, ILO, SIL and TEZ, respectively.

### Measurement protocol and bronchoprovocation challenges

Four weeks following the surgical preparation of ACS, the rats were again anaesthetized and intubated, and the experimental protocol was performed. EELV was first measured while the rats were breathing spontaneously. A femoral vein was then cannulated for drug delivery. When stable hemodynamic and respiratory mechanical conditions had been reached, a set of Zrs data including 4-6 6-s long recordings was recorded under the baseline conditions. Increasing doses of iv methacholine (MCh) were then administered at doses of 2, 4, 8, 12 and 24 μg/kg/min, and at each infusion rate 3-5 Zrs recordings were made during the development of steady-state bronchoconstriction (usually 3-5 min after the onset of MCh). The equivalent dose causing a 50% increase in Raw (ED_50_) was calculated in each rat by linear interpolation. Following these measurements, Ppa was measured as described above.

### Statistical analyses

The scatters in the parameters were expressed by the SE values. The Kolmogorov-Smirnov test was used to test data for normality. One-way analysis of variances (ANOVA) was performed to test significant differences in the parameters among the groups. Two-way repeated measures ANOVA, with variables of treatment (i.e. the protocol groups) and MCh dose, was used to evaluate the effects of the various types of medication on the precapillary PHT and subsequent lung responsiveness to the constrictor challenge. Pairwise comparisons were performed by means of Student-Newman-Keuls multiple comparison procedures. Pearson correlation test was used to assess the strength of associations between variables. Statistical tests were carried out with the significance level set at p < 0.05.

## Results

There was no significant difference in body weight among the protocol groups either at the beginning of the study (ranging from 340 ± 9 g to 360 ± 4 g, p = 0.11) or at the time of the experiments 4 weeks later (ranging from 413 ± 14 g to 454 ± 12 g, p = 0.1).

**Figure 2:**
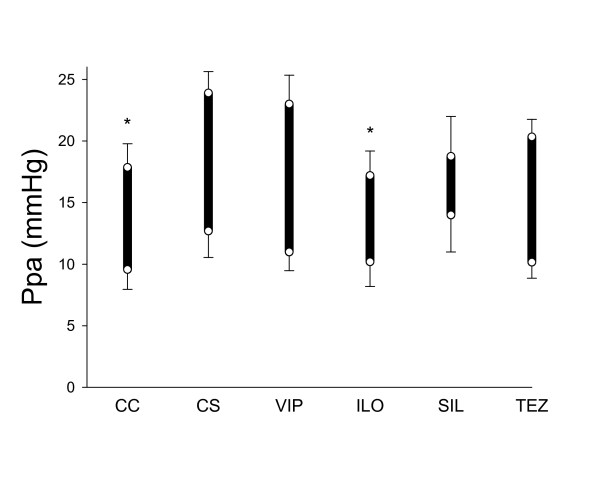
**Systolic (top of the bars) and diastolic (bottom of the bars) pulmonary arterial pressures in the rats involved in the different groups**. CC: control group, CS: shunt, no treatments, VIP: shunt, treatment with VIP, ILO: shunt, treatment with iloprost, SIL: shunt, treatment with sildenafil, TEZ: shunt, treatment with tezosentan. *: p < 0.05 vs the data obtained in the rats with precapillary pulmonary hypertension with no drug treatment (Group CS).

Figure 2 demonstrates the systolic and diastolic values of Ppa for the various groups. Preparation of the ACS led to a significant increase in systolic Ppa (p = 0.036). This PHT was significantly inhibited only by iloprost (p = 0.035); none of the other treatments affected the alteration in Ppa. No change in the diastolic Ppa was detected (p = 0.15).

**Figure 3:**
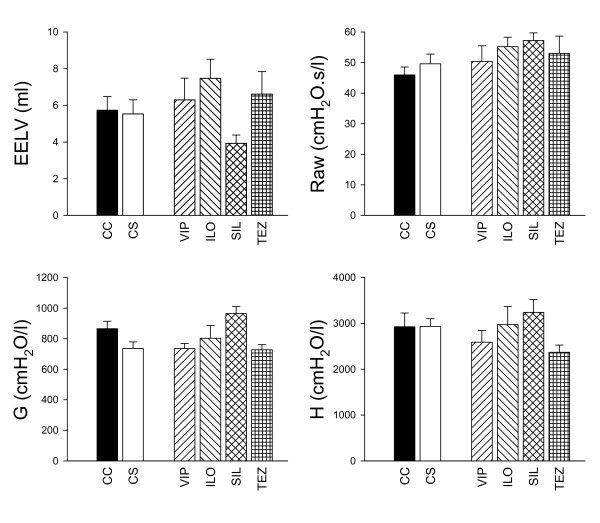
**The baseline end-expiratory lung volume (EELV), airway resistance (Raw), respiratory tissue damping (G) and elastance (H) data obtained in the different groups**. CC: control group, CS: shunt, no treatments, VIP: shunt, treatment with VIP, ILO: shunt, treatment with iloprost, SIL: shunt, treatment with sildenafil, TEZ: shunt, treatment with tezosentan.

The baseline in the EELV and the forced oscillatory parameters in the different groups are displayed in Figure 3. ACS preparation and subsequent treatments did not affect the basal lung volume (p = 0.22), the basal Raw (p = 0.28) or the respiratory tissue parameters (p = 0.45).

**Figure 4:**
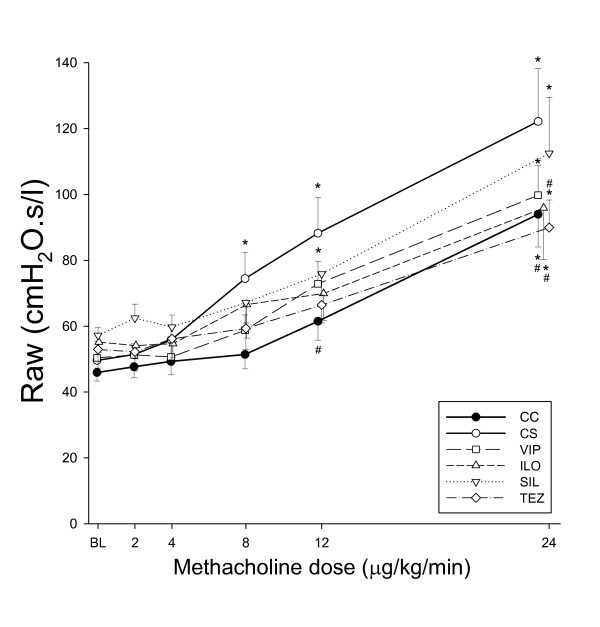
**Methacholine-induced changes in airway resistance (Raw) in the different groups**. CC: control group, CS: shunt, no treatments, VIP: shunt, treatment with VIP, ILO: shunt, treatment with iloprost, SIL: shunt, treatment with sildenafil, TEZ: shunt, treatment with tezosentan. *: p < 0.05 vs the value obtained under baseline conditions (BL) within a group; #: p < 0.05 vs. the data obtained in the rats with precapillary pulmonary hypertension without drug treatment (Group CS).

Figure 4 depicts the airway responses to MCh challenges in the groups. MCh induced a dose-dependent bronchoconstriction of various magnitude in all groups. ACS preparation led to the development of BHR, as illustrated by the significant upward shift in the dose response-curve. Each of the drug treatments exerted a protective effect against this hyperresponsiveness to MCh. The MCh-induced changes in G and H were far more moderate and did not exhibit a statistically significant difference among the groups (p = 0.45, and p = 0.26 for G and H, respectively; Table 1).

**Table 1:** Respiratory tissue parameters under baseline conditions, during MCh infusion at a dose of 24 μg/kg/min, and their percentage changes in the different groups

	**G (cmH**_**2**_**O/l)**		Change in G	**H (cmH**_**2**_**O/l)**		Change in H
	
	Baseline	MCh 24 μg/kg/min	%	Baseline	MCh 24 μg/kg/min	%
CC	866 ± 49	1150 ± 76*	33 ± 5	2927 ± 299	3767 ± 334*	31 ± 4
CS	736 ± 42	968 ± 78*	31 ± 5	2936 ± 167	3329 ± 195*	15 ± 6
VIP	735 ± 33	991 ± 49*	37 ± 10	2589 ± 258	3139 ± 231*	24 ± 5
ILO	802 ± 84	930 ± 65*	21 ± 9	2972 ± 397	3061 ± 147	10 ± 10
SIL	964 ± 45	1159 ± 50*	21 ± 6	3240 ± 281	3741 ± 278*	16 ± 3
TEZ	727 ± 34	911 ± 58*	25 ± 3	2370 ± 156	3001 ± 232*	26 ± 2

CC: control group, CS: shunt, no treatments, VIP: shunt, treatment with VIP, ILO: shunt, treatment with iloprost, SIL: shunt, treatment with sildenafil, TEZ: shunt, treatment with tezosentan.

*: p < 0.05 vs. the value obtained under baseline conditions within a group.

**Figure 5:**
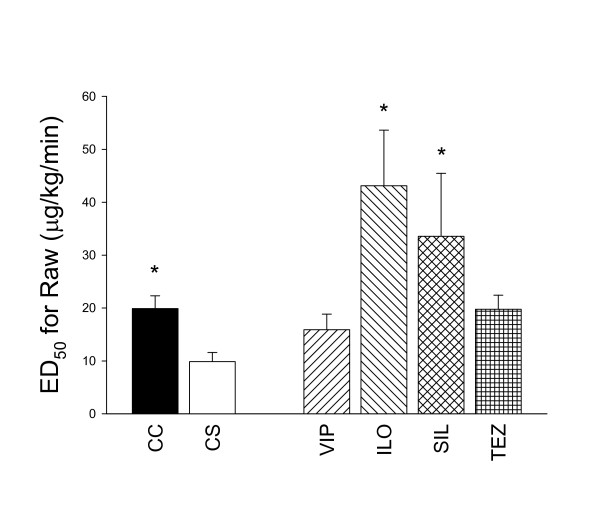
**Equivalent dose required to cause a 50% increase in airway resistance (ED_50_) in the different groups**. CC: control group, CS: shunt, no treatments, VIP: shunt, treatment with VIP, ILO: shunt, treatment with iloprost, SIL: shunt, treatment with sildenafil, TEZ: shunt, treatment with tezosentan. *: p < 0.05 vs. the data obtained in the rats with precapillary pulmonary hypertension without drug treatment (Group CS).

To illustrate the effects of the various treatments on the airway responses to MCh, the ED_50 _values are compared in Figure 5. The BHR induced by generating a precapillary PHT by ACS was manifested in a significant decrease in ED_50 _in Group CS relative to that in Group CC (p = 0.008). All of the treatments exerted a significant protective potential, leading in Groups VIP and TEZ an airway responsiveness similar to that observed in the sham operated rats (Group CC). This parameter revealed even overprotection of the airway responsiveness to MCh in Group ILO and Group SIL.

**Figure 6:**
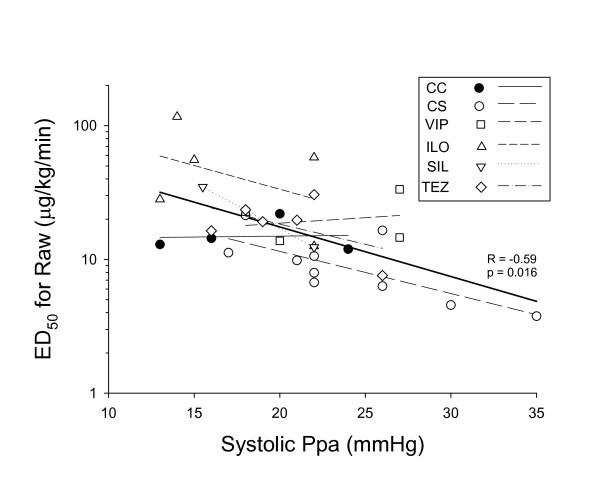
**The relationships between the systolic pulmonary arterial pressure (Ppa) and the lung responsiveness expressed as the equivalent dose required to cause a 50% increase in the airway resistance (ED_50_) in the different groups **. CC: control group, CS: shunt, no treatments, VIP: shunt, treatment with VIP, ILO: shunt, treatment with iloprost, SIL: shunt, treatment with sildenafil, TEZ: shunt, treatment with tezosentan. Symbols: individual data points in each rat, thin lines: regressions for individual groups, thick line: linear regression for the pooled data.

The relationships between the systolic Ppa and ED_50 _is depicted in Figure 6. These parameters relating to the pulmonary haemodynamics and lung responsiveness exhibited a statistically significant correlation for the entire study population (R = -0.59, p = 0.016) and were variable for the individual study groups with closest correlation being observed in Group CS (R = -0.6) and the weakest in rats with no intervention (Group C, R = 0.1).

## Discussion

Various treatment strategies applied against PHT act on the imbalance between the vasoactive constrictor-proliferative and vasorelaxing-antiproliferative mediators. In the present study, we investigated whether these treatments provide protection against the enhanced airway responsiveness observed in the presence of precapillary PHT. The results obtained in this well-validated animal model of BHR originating from precapillary PHT induced by ACS preparation demonstrate that all the treatment modes studied, including the action on the VIP, PC, NO and ET-1 pathways, exhibited a beneficial profile in preventing the development of BHR. Pooling of all the data points obtained from the groups revealed that the systolic Ppa level and the magnitude of the lung responsiveness were associated.

It was earlier reported [11] that the creation of an ACS led to precapillary PHT, which was associated with enhanced airway responsiveness to MCh. This experimental model has been extensively validated previously both by ourselves and by other research groups, and has been proved to provide an increased pulmonary blood flow leading to PHT [11-13,20]. Precise measurement of the pulmonary haemodynamics in rats was complicated by the small size of the animal and the lack of catheter-tip balloon, which normally helps to introduce to the pulmonary artery. Due to this technical difficulty, technically acceptable Ppa recordings were possible only in about two-third of the rats. According to literature [11,13,20], we also noted an elevated systolic Ppa in the rats in Group CS, confirming the validity of the experimental model for investigation of the pulmonary consequences of chronic precapillary PHT. It is noteworthy, however, that the model applied here mimics clinical conditions encountered in patients with left-to-right shunts and increased pulmonary blood flow, and the results may not be extrapolated to other forms of PHT. The altered mechanical interdependence between the pulmonary vasculature and the respiratory tract is responsible for the BHR in this experimental model, thus the effects of the applied treatment strategies in the present study are valid for this pulmonary haemodynamical abnormality.

In agreement with previous data [11], we did not observe statistically significant changes in the baseline values of the respiratory mechanical parameters following sustained precapillary PHT. However, normalization of the Raw to the lung volume revealed a statistically significant increase in this specific Raw (p < 0.006), which is in harmony with the earlier results that ACS causes a detectable elevation only in the corrected baseline Raw. Accordingly, it can be anticipated that the experimental model applied here reflects the major features of the respiratory mechanical changes encountered under baseline conditions in patients with chronic precapillary PHT.

The three major mechanisms involved in the development of PHT are the PC, the ET and NO pathways. The pathological changes in these pathways are manifested by an imbalance of the mediators with vasodilation or vasoconstriction potential. These pathways have also been implicated in the regulation of the airway smooth muscle tone [8,10,21,22]. Thus, we restored an optimum balance between the constrictor/dilator mediators through selective targeting of these mechanisms to characterize whether these treatment strategies affect lung responsiveness.

Iloprost is a PC analogue and a potent pulmonary vasodilator that exerts its effect through the activation of cyclic adenosine monophosphate (cAMP) [2]. Since the molecule has a short half-life (approximately 20 min), we followed the common strategy of delivering it via a continuous infusion. In agreement with previous findings, this treatment strategy effectively inhibited the PHT [23,24]. Over and above this well-established beneficial pulmonary vascular effect, our study is the first to demonstrate that this pharmacotherapeutic management is associated with complete prevention of the development of BHR.

Another essential pathway involved in the pathogenesis of PHT is related to a dysfunction in the key pulmonary vasodilator NO [25]. Enhancement of the NO-mediated effects by phosphodiesterase 5 inhibitors such as sildenafil has therefore been proposed as an effective mode of treatment against PHT [9,26]. NO is known to exert a potent bronchodilation effect [27], and its overexpression leads to a direct relaxing effect on the airway smooth muscle. Since excessive NO in the lungs has been shown to inhibit MCh-induced bronchoconstriction [28], this mechanism is probably involved in the complete prevention of BHR in Group SIL in the present study.

The rationale of testing the ability of tezosentan, a dual ET-1 receptor antagonist, to prevent BHR emerged from the crucial involvement of ET-1 in the pathogenesis of PHT [25]. ET-1 exerts its regulatory effect on the pulmonary vascular and bronchial tone via two receptor subtypes (ET_A _and ET_B_). The roles of these receptor subtypes on the pulmonary vascular and bronchial tone are dissociated, with ET_A _stimulation leading to vasoconstriction and bronchodilation [10,29], while activation of ET_B _receptors results in the liberation of vasodilator mediators with a concomitant bronchodilation potential [10,30]. The opposing effects of ET_A _and ET_B _following dual receptor blockade may therefore have resulted in the lack of significant change in the Ppa in the present study. However, the similar abilities of ET_A _and ET_B _to relax the airway smooth muscle are reflected in the potential of tezosentan to prevent BHR effectively. This raises the unsolved controversy of the use of specific ET_A _or dual blockers: clinical studies have so far failed to reveal significant differences between selective ET_A _and dual receptor blockade [31]. However, our data suggest that the detrimental effects of precapillary PHT can be effectively prevented by dual ET-1 receptor blockade.

VIP plays a major role in regulating the smooth muscle tone around both the pulmonary vasculature and the bronchi via the non-adrenergic non-cholinergic nervous system [32-34]. Since VIP exerts its relaxation potential on the smooth muscle by opening Ca^2+^-activated K^+ ^channels through a cAMP-dependent mechanism [35,36] and by inducing NO release from VIP/NO-containing nerve fibers and the endothelium [32,37], its inhalation has recently been advocated in the treatment of PHT [38,39]. Although previous studies have suggested the efficacy of this peptide in inducing transient pulmonary vasodilation in another model of PHT [39], our findings indicate that the chronic continuous intraperitoneal administration of VIP did not provide a decrease in Ppa in the presence of precapillary PHT. However, BHR was eliminated by VIP treatment, which is in accord with previous results demonstrating its bronchodilation properties [10,40].

### Summary and conclusions

This comparison of treatment strategies aimed at reestablishment of the balance between the various smooth muscle constrictor and relaxation mediators revealed similar abilities of the agonists acting on the PC, NO, ET-1 and VIP pathways to protect the development of airway hyperreactivity subsequent to precapillary PHT. The pathophysiological mechanisms responsible for the fairly uniform bronchial effects of the various treatments may be inferred from the significant correlation between the lung responsiveness and the level of systolic PHT (Figure 6). This close association suggests that independently of the pharmacotherapeutical approach to influence the pulmonary vasculature, it is the level of Ppa which determines the lung responsiveness through the existence of a close mechanical interdependence between the pulmonary vasculature and the airway tree.

## Competing interests

The authors declare that they have no competing interests.

## Authors' contributions

WH conducted the design of the study and had a major role in drafting the manuscript. GA and TJ carried out the experiments and the preliminary data analyses. FF performed the surgical preparation and helped in conducting the pretreatments and performing the experiments. BSU participated in the study design, data collection and helped in processing the data. MB participated in the design of the study and interpretation of the experimental findings. FP coordinated the various experimental approaches, contributed in their design and in the manuscript preparation. All authors read and approved the final manuscript.

## References

[B1] Hoeper MM Rubin LJ Update in pulmonary hypertension 2005 Am J Respir Crit Care Med 2006 173 499 505 10.1164/rccm.2512003 16493163

[B2] McLaughlin VV Genthner DE Panella MM Rich S Reduction in pulmonary vascular resistance with long-term epoprostenol (prostacyclin) therapy in primary pulmonary hypertension N Engl J Med 1998 338 273 277 10.1056/NEJM199801293380501 9445406

[B3] Murali S Pulmonary arterial hypertension Curr Opin Crit Care 2006 12 228 234 10.1097/01.ccx.0000224867.78581.0c 16672782

[B4] Rich S The current treatment of pulmonary arterial hypertension: time to redefine success Chest 2006 130 1198 1202 10.1378/chest.130.4.1198 17035456

[B5] Humbert M Sitbon O Simonneau G Treatment of pulmonary arterial hypertension N Engl J Med 2004 351 1425 1436 10.1056/NEJMra040291 15459304

[B6] Howard LS Morrell NW New therapeutic agents for pulmonary vascular disease Paediatr Respir Rev 2005 6 285 291 10.1016/j.prrv.2005.09.006 16298312

[B7] Keith IM The role of endogenous lung neuropeptides in regulation of the pulmonary circulation Physiol Res 2000 49 519 537 11191357

[B8] Habre W Petak F Ruchonnet-Metrailler I Donati Y Tolsa JF Lele E Albu G Beghetti M Barazzone-Argiroffo C The role of endothelin-1 in hyperoxia-induced lung injury in mice Respir Res 2006 7 45 10.1186/1465-9921-7-45 16566828 PMC1475846

[B9] Ghofrani HA Osterloh IH Grimminger F Sildenafil: from angina to erectile dysfunction to pulmonary hypertension and beyond Nat Rev Drug Discov 2006 5 689 702 10.1038/nrd2030 16883306 PMC7097805

[B10] Janosi T Petak F Fontao F Morel DR Beghetti M Habre W Differential roles of endothelin-1 ETA and ETB receptors and vasoactive intestinal polypeptide in regulation of the airways and the pulmonary vasculature in isolated rat lung Exp Physiol 2008 93 1210 1219 10.1113/expphysiol.2008.042481 18567602

[B11] von Ungern-Sternberg BS Habre W Regli A Pache JC Fontao F Janosi TZ Beghetti M Petak F Precapillary pulmonary hypertension leads to reversible bronchial hyperreactivity in rats Exp Lung Res 2010 36 129 139 10.3109/01902140903214667 20334605

[B12] Garcia R Diebold S Simple, rapid, and effective method of producing aortocaval shunts in the rat Cardiovasc Res 1990 24 430 432 10.1093/cvr/24.5.430 2142618

[B13] Ocampo C Ingram P Ilbawi M Arcilla R Gupta M Revisiting the surgical creation of volume load by aorto-caval shunt in rats Mol Cell Biochem 2003 251 139 143 10.1023/A:1025450403668 14575315

[B14] Habre W Janosi TZ Fontao F Meyers C Albu G Pache JC Petak F Mechanisms for lung function impairment and airway hyperresponsiveness following chronic hypoxia in rats Am J Physiol Lung Cell Mol Physiol 2010 298 L607 614 10.1152/ajplung.00222.2009 20139180

[B15] Janosi TZ Adamicza A Zosky GR Asztalos T Sly PD Hantos Z Plethysmographic estimation of thoracic gas volume in apneic mice J Appl Physiol 2006 101 454 459 10.1152/japplphysiol.00011.2006 16645196

[B16] Petak F Hantos Z Adamicza A Asztalos T Sly PD Methacholine-induced bronchoconstriction in rats: effects of intravenous vs. aerosol delivery J Appl Physiol 1997 82 1479 1487 10.1063/1.365927 9134896

[B17] Petak F Habre W Hantos Z Sly PD Morel DR Effects of pulmonary vascular pressures and flow on airway and parenchymal mechanics in isolated rat lungs J Appl Physiol 2002 92 169 178 11744657 10.1152/jappl.2002.92.1.169

[B18] Hantos Z Daroczy B Suki B Nagy S Fredberg JJ Input impedance and peripheral inhomogeneity of dog lungs J Appl Physiol 1992 72 168 178 10.1063/1.352153 1537711

[B19] Petak F Hall GL Sly PD Repeated measurements of airway and parenchymal mechanics in rats by using low-frequency oscillations J Appl Physiol 1998 84 1680 1686 10.1063/1.368257 9572817

[B20] Lam CF Peterson TE Croatt AJ Nath KA Katusic ZS Functional adaptation and remodeling of pulmonary artery in flow-induced pulmonary hypertension Am J Physiol Heart Circ Physiol 2005 289 H2334 2341 10.1152/ajpheart.00375.2005 15964923

[B21] Larsen GL Loader J Fratelli C Kang JK Dakhama A Colasurdo GN Modulation of airway responses by prostaglandins in young and fully grown rabbits Am J Physiol Lung Cell Mol Physiol 2007 293 L239 244 10.1152/ajplung.00413.2006 17483193

[B22] Ricciardolo FL Sterk PJ Gaston B Folkerts G Nitric oxide in health and disease of the respiratory system Physiol Rev 2004 84 731 765 10.1152/physrev.00034.2003 15269335

[B23] Higenbottam TW Butt AY Dinh-Xaun AT Takao M Cremona G Akamine S Treatment of pulmonary hypertension with the continuous infusion of a prostacyclin analogue, iloprost Heart 1998 79 175 179 9538312 10.1136/hrt.79.2.175 PMC1728597

[B24] Gomberg-Maitland M Olschewski H Prostacyclin therapies for the treatment of pulmonary arterial hypertension Eur Respir J 2008 31 891 901 10.1183/09031936.00097107 18378784

[B25] Giaid A Yanagisawa M Langleben D Michel RP Levy R Shennib H Kimura S Masaki T Duguid WP Stewart DJ Expression of endothelin-1 in the lungs of patients with pulmonary hypertension N Engl J Med 1993 328 1732 1739 10.1056/NEJM199306173282402 8497283

[B26] Galie N Ghofrani HA Torbicki A Barst RJ Rubin LJ Badesch D Fleming T Parpia T Burgess G Branzi A Sildenafil citrate therapy for pulmonary arterial hypertension N Engl J Med 2005 353 2148 2157 10.1056/NEJMoa050010 16291984

[B27] Brown RH Zerhouni EA Hirshman CA Reversal of bronchoconstriction by inhaled nitric oxide. Histamine versus methacholine Am J Respir Crit Care Med 1994 150 233 237 8025755 10.1164/ajrccm.150.1.8025755

[B28] Hogman M Frostell C Arnberg H Hedenstierna G Inhalation of nitric oxide modulates methacholine-induced bronchoconstriction in the rabbit Eur Respir J 1993 6 177 180 8444288

[B29] MacLean MR McCulloch KM Baird M Endothelin ETA- and ETB-receptor-mediated vasoconstriction in rat pulmonary arteries and arterioles J Cardiovasc Pharmacol 1994 23 838 845 10.1097/00005344-199405000-00022 7521470

[B30] Leuchte HH Meis T El-Nounou M Michalek J Behr J Inhalation of endothelin receptor blockers in pulmonary hypertension Am J Physiol Lung Cell Mol Physiol 2008 294 L772 777 10.1152/ajplung.00405.2007 18296497

[B31] Opitz CF Ewert R Kirch W Pittrow D Inhibition of endothelin receptors in the treatment of pulmonary arterial hypertension: does selectivity matter? Eur Heart J 2008 29 1936 1948 10.1093/eurheartj/ehn234 18562303 PMC2515885

[B32] Anaid S Petkov V Baykuscheva-Gentscheva T Hoeger H Painsipp E Holzer P Mosgoeller W Involvement of endothelial NO in the dilator effect of VIP on rat isolated pulmonary artery Regul Pept 2007 139 102 108 10.1016/j.regpep.2006.10.012 17174416

[B33] Iwabuchi S Ono S Tanita T Koike K Fujimura S Vasoactive intestinal peptide causes nitric oxide-dependent pulmonary vasodilation in isolated rat lung Respiration 1997 64 54 58 10.1159/000196643 9044476

[B34] Crimi N Palermo F Oliveri R Palermo B Vancheri C Polosa R Mistretta A Effect of vasoactive intestinal peptide (VIP) on propranolol-induced bronchoconstriction J Allergy Clin Immunol 1988 82 617 621 10.1016/0091-6749(88)90973-6 2971708

[B35] Hagen BM Bayguinov O Sanders KM VIP and PACAP regulate localized Ca2+ transients via cAMP-dependent mechanism Am J Physiol Cell Physiol 2006 291 C375 385 10.1152/ajpcell.00495.2005 16571863

[B36] Kishi M Takeuchi T Suthamnatpong N Ishii T Nishio H Hata F Takewaki T VIP- and PACAP-mediated nonadrenergic, noncholinergic inhibition in longitudinal muscle of rat distal colon: involvement of activation of charybdotoxin- and apamin-sensitive K+ channels Br J Pharmacol 1996 119 623 630 8904634 10.1111/j.1476-5381.1996.tb15719.x PMC1915760

[B37] Lee TJ Nitric oxide and the cerebral vascular function J Biomed Sci 2000 7 16 26 10.1007/BF02255914 10644885

[B38] Petkov V Mosgoeller W Ziesche R Raderer M Stiebellehner L Vonbank K Funk GC Hamilton G Novotny C Burian B Block LH Vasoactive intestinal peptide as a new drug for treatment of primary pulmonary hypertension J Clin Invest 2003 111 1339 1346 12727925 10.1172/JCI17500 PMC154449

[B39] Leuchte HH Baezner C Baumgartner RA Bevec D Bacher G Neurohr C Behr J Inhalation of vasoactive intestinal peptide in pulmonary hypertension Eur Respir J 2008 32 1289 1294 10.1183/09031936.00050008 18978135

[B40] Barnes PJ Dixon CM The effect of inhaled vasoactive intestinal peptide on bronchial reactivity to histamine in humans Am Rev Respir Dis 1984 130 162 166 6465669 10.1164/arrd.1984.130.2.162

